# A classification model to predict synergism/antagonism of cytotoxic mixtures using protein-drug docking scores

**DOI:** 10.1186/1471-2210-8-13

**Published:** 2008-07-29

**Authors:** John C Boik, Robert A Newman

**Affiliations:** 1 Department of Experimental Therapeutics, University of Texas M. D. Anderson Cancer Center, 8000 El Rio, Houston, TX 77054, USA

## Abstract

**Background:**

Safer and more effective mixtures of anticancer drugs are needed, and modeling can assist in this endeavor. This paper describes classification models that were constructed to predict which fixed-ratio mixtures created from a pool of 10 drugs would show a high degree of *in-vitro *synergism against H460 human lung cancer cells. One of the tested drugs was doxorubicin and the others were natural compounds including quercetin, curcumin, and EGCG. Explanatory variables were based on virtual docking profiles. Docking profiles for the 10 drugs were obtained for 1087 proteins using commercial docking software. The cytotoxicity of all 10 drugs and of 45 of the 1,013 possible mixtures was tested in the laboratory and synergism indices were generated using the MixLow method. Model accuracy was assessed using cross validation, as well as using predictions on a new set of 10 tested mixtures. Results were compared to models where explanatory variables were constructed using the pseudomolecule approach of Sheridan.

**Results:**

On this data set, the pseudomolecule and docking data approach produce models of similar accuracy. Leave-one-out precision for the negative (highly synergistic) class and the positive (low- or non-synergistic) class was 0.73 and 0.80, respectively. Precision for a nonstandard leave-many-out cross validation procedure was 0.60 and 0.77 for the negative and positive classes, respectively.

**Conclusion:**

Useful classification models can be constructed to predict drug synergism, even in those situations where a limited subset of component drugs can be tested. Compared to the pseudomolecule approach, the virtual docking approach has the advantage of greater potential for biologic interpretation. This distinction may become important as virtual docking software becomes more accurate and docking results more closely resemble actual binding affinities. This is the first published report of a model designed to predict the degree of in-vitro synergism based on the pseudomolecule or docking data approach.

## Background

Combination chemotherapy for cancer was introduced in the 1960s [1,2] as a means to increase the efficacy of anticancer drugs, avoid problems with drug resistance, and/or reduce adverse effects. While today almost all anticancer drugs are administered in combinations, or mixtures, an urgent need remains to develop mixtures that are more effective and safe. The mixture development process typically occurs after individual drugs are approved for clinical use. (Approval refers to market approval by the United States Food and Drug Administration (US-FDA)). As such, the pool of drugs that is available for creating mixtures is small compared to a pool that also includes unapproved but potentially useful compounds. By incorporating mixture design early in the preclinical phase of development and by considering all potentially useful components regardless of their approval status, more opportunities exist to optimize mixture action. A greater number and variety of candidate compounds should allow increased flexibility and control with regard to affecting therapeutic target(s). In the remainder of this paper, the term *drug *is used to refer to both approved compounds and potentially useful compounds.

In addition to using a larger pool of candidate drugs, it is of interest to consider large mixture sizes as an aid to gaining greater control over mixture action. Typically, most mixtures used in the clinic contain two to five cytotoxic drugs. The tendency to limit mixture size to this range is due in large part to concerns over overlapping toxicity profiles. One can speculate, however, that if some drugs in a mixture were of low systemic toxicity but still somewhat cytotoxic to cancer cells (not necessarily strongly cytotoxic) a larger number of drugs could be safely used. If the inclusion of such drugs improved mixture efficacy via synergism, then the larger mixtures might be clinically useful.

One difficulty that arises with large candidate pools and large mixture sizes, however, is the combinatorial explosion of mixtures that can be created. For *n *drugs, 2^*n*^-*n*-1 fixed-ratio mixtures of two or more drugs can be created. From a pool of 10 drugs, 1,013 mixtures are possible. The problem is considerably aggravated if ratios between drugs in a mixture are allowed to freely vary. For practical reasons, this study was limited to a pool of ten drugs, with fixed concentration ratios used between drugs. Ratios were based on relative IC50 values. Mixtures consisted of doxorubicin and one or more of nine natural compounds, with the later chosen from a pool of 115,000 natural compounds. Criteria for choosing the nine included commercial availability, a prediction of low systemic toxicity in rats, a prediction of modest or stronger *in-vitro *cytotoxicity in multiple NCI cell lines, and activity in the cytotoxicity assay used here. Thus the nine compounds are thought to be relatively non-toxic to mammals but still cytotoxic to cancer cells at reasonably low concentrations. Indeed, several of the compounds, including curcumin, quercetin, and EGCG, are regularly ingested by humans in the diet.

The use of fixed ratios resulted in 1,013 mixtures, which are still too numerous to comprehensively test. Therefore, the goal of this study was to develop a modeling approach that would be useful in predicting which mixtures are likely to be highly synergistic. Classification models for drug interaction were developed and trained on a small fraction of the 1,013 possible mixtures. The training set consisted of 45 mixtures, or about four percent of the total sample space.

The responses modeled were derived from confidence intervals of a Loewe-additivity drug interaction index that was estimated using the MixLow method [3]. To account for concentration-dependent changes in interactions, a function of the interaction index was integrated over a moderate range of fraction-affected values (see Methods). Thus, responses represented the degree of synergism or antagonism averaged over a moderate range of mixture concentrations. In the remaining text, responses will sometimes be referred to as *synergism scores*.

One or more of three sets of explanatory variables were used to construct the models. One set was comprised of binary indicators of mixture composition – where a zero indicated that a drug was not in a mixture, and a one indicated otherwise. There are at least two drawbacks to models based only on this set of variables, however. First, the models would not be able to make predictions for mixtures containing drugs that the models had not been trained on. Second, the explanatory variables do not contain direct biologic information and so biologic interpretation of the results is limited.

A major challenge in devising other sets of explanatory variables is to find suitable methods for encoding the characteristics of a mixture into a form that can be manipulated mathematically. One approach, introduced by Sheridan [4], is to develop quantitative structure-activity relationship (QSAR) models using "pseudomolecules" to represent mixtures. Pseudomolecules represent the average of molecular descriptors over all component drugs. Using this approach, explanatory variables for a given mixture might include the average number of nitrogen atoms over all drugs in the mixture, the average molecular weight, and so on. One drawback to the pseudomolecule approach is that like composition data, the information contained in pseudomolecules does not have direct biological meaning. The pseudomolecule approach was used to construct the second set of explanatory variables. This is the first time the approach has been used to predict drug synergism.

The third set of explanatory variables is constructed using a new approach whereby mixtures are (ideally) represented as a function of predicted protein binding patterns of component drugs. Most drugs influence cellular activity by binding to one or more proteins and therefore a mixture's activity should be dependent on the protein-binding characteristics of the component drugs. By using protein-drug binding data as explanatory variables, a systems biology frame of reference might be gained.

Unfortunately, protein-drug binding data are expensive to generate in the laboratory. One alternative is to use docking scores generated by virtual docking software. Unfortunately, state-of-the-art virtual docking programs cannot predict protein-drug binding affinity with high accuracy [5]. Docking programs can, however, be useful for classifying drugs into high and low affinity categories, although high rates of false-positives remain a common problem [6]. (False positives are easier to identify, as data for identifying false negatives is generally not available.) Despite their limitations, virtual docking scores are used in this paper. A set of explanatory variables was created based on docking scores generated by the commercial docking program Ehits [7]. Docking was conducted for 10 selected drugs and 1,087 proteins whose structures were obtained from the Protein Data Bank (PDB). Of these, 286 proteins were successfully docked to all 10 drugs and were predicted to bind strongly with at least one of them.

Protein-drug docking scores have already been used as substitutes for molecular descriptors in QSAR models [8,9]. In these studies, however, models were constructed for single drugs. A means to use docking scores for modeling mixtures has not yet been developed. A straightforward method is proposed here in which scores are first converted to binary values. A value of one is assigned to any protein-drug combination for which the docking score is both below a low threshold and below that calculated for the co-crystallized ligand – low docking scores are associated with a higher chance of binding. A value of zero is assigned otherwise. Next, mixture-protein scores are assigned by counting the number of drugs in a mixture that are predicted to bind to a given protein. The hypothesis is that the effects of a mixture may be related to how many of the component drugs bind to individual proteins. If many drugs in a mixture bind to a given protein, the chance of inhibiting the protein may be greater than if none or only a few drugs bind. Because each column in the explanatory data matrix corresponds to count data for one protein, and models use multiple explanatory data columns, the models should ideally be able to identify relationships between synergism scores and inhibition of multiple proteins (i.e., proteins on multiple or single pathways). A hypothetical example of calculating mixture-protein scores is presented in Table 1. Informative reviews of the relationships between protein networks, multi-target therapies, and synergism were published by Araujo et al. [10] and Zimmermann et al. [11]. For the remainder of this paper, the mixture-protein scores are referred to as *docking data*.

**Table 1:** Example Calculations for Mixture Scores

**1. Obtain Docking Scores**	Protein 1	Protein 2
Drug 1	-5.5	-2.1
Drug 2	-3.1	-2.2
Drug 3	-1.2	-4.3
**2. Assign binary values using threshold of -3.0**	Protein 1	Protein 2
Drug 1	1	0
Drug 2	1	0
Drug 3	0	1
**3. Sum binary values**	Protein 1	Protein 2
Mixture 1 (Drug 1 + Drug 2)	2	0
Mixture 2 (Drug 2 + Drug 3)	1	1

As noted above, docking software is not able to predict binding affinity with high precision. Even though the docking scores are used here only to classify the drugs into high and low affinity groups, it is highly likely that some drugs are misclassified. Using current software, the degree to which the derived docking data is an accurate reflection of true binding affinity is uncertain. At worst the derived docking data is unrelated to binding affinity and must be viewed simply as a set of mathematical descriptors that may possess discriminative ability. At best they modestly reflect true binding affinity and therefore possess some biologic meaning. To be conservative it is prudent to consider the current docking data simply as mathematical descriptors. As docking software improves, however, the approach outlined here should be better able to generate descriptors with true biologic meaning.

Leave-one-out and leave-many-out cross validation was used to assess the accuracy of models constructed here. Results based on docking scores were contrasted with results based on pseudomolecule data. In addition, a regression model was constructed using docking scores and the model was used to make predictions for all 1,013 possible mixtures. From these results an additional 10 mixtures were selected for testing. Synergism scores obtained from these experiments were used to create an additional test set for the classification model. Lastly, models were constructed using pseudomolecule and docking data where synergism scores were scrambled. Overall, results suggest that accuracies of the pseudomolecule and docking-data models were similar. A larger training set would be needed to better determine if one method is superior to the other. In addition, both models performed significantly worse on scrambled responses, indicating that the relationships found were not due to chance alone. This paper presents a new method to generate discriminative descriptors for mixture models and to our knowledge is the first published report of a predictive model for drug synergism based on virtual docking data. Using a different approach and a yeast proliferation assay, Lehar et al. [12] have produced a model to predict the type of synergism (Loewe, Bliss, etc.) based on the type of protein interaction (sterol pathway single protein target, sterol pathway different target proteins, and proteins on other pathways). Their method also appeared useful for cytotoxicity data. To use the model, drug targets must be known.

## Results

### IC50 values of drugs and mixtures and synergism scores

IC50 values and their standard errors (SE), along with synergism scores (responses) are listed in Table S.1 of Additional File 1. For the reader's convenience, IC50 values for single drugs are given in units of both μL and μg/ml (see Methods section for an explanation of units).

Mixture composition was based on preliminary ratios of IC50 values between the 10 drugs. These ratios differed only slightly from the IC50 values listed in Table S.1. A list of mixtures and their compositions is given in Table S.2 of Additional File 1.

### Classification models of drug interaction

A classification model was constructed using only the docking data as explanatory variables. The model was assessed by a nonstandard leave-many-out cross validation (CV) procedure in which each CV training set included all mixtures except those that contained a specified drug. The corresponding CV test sets consisted of all mixtures that did contain the specified drug. In this way, models were used to make predictions on mixtures that contained a drug the models had not been trained on. In practice, it is desirable to have an accurate predictive model that is trained using only a subset of candidate drugs. To assess this capability, the nonstandard leave-many out procedure was used rather than a standard one where assignment of mixtures to training sets is done randomly.

Note that by design the leave-many-out procedure created challenging CV training/testing sets. First, only 26 of the 45 examples were used in a given CV training set, on average. Second, as already mentioned, the CV test sets were constructed of mixtures that contained a drug the model had not been trained on.

Because a given drug appeared in several mixtures, each mixture appeared in several different CV test sets. As such, the total number of predictions made on all CV test sets was 177, not 45. Rather than form a consensus prediction for each mixture across all CV test sets (which would tend to increase model accuracy), all 177 predictions were used in assessing model quality.

Precision for the docking-data model was 0.77 on the positive labels and 0.60 on the negative ones. Relative to other CV testing sets, predictions for mixtures in the doxorubicin hold-out set were poor – precision was 1.0 on positive labels and 0.08 on negative ones. Excluding these 19 predictions, the precision was 0.76 on both the positive and negative labels.

The feature selection algorithm for this model identified about 35 columns of explanatory variables as being important, depending on the training set. Across all cross-validation models, the ten most common proteins selected during feature selection were 1PXJ (cyclin dependent kinase 2), 1JYX (beta-galactosidase), 1YTA (oligoribonuclease), 1NAI (UDP-galactose 4-epimerase), 2H42 (phosphodiesterase 5), 17GS (glutathione S-transferase), 2ITM (xylulose kinase), 1XOQ (phosphodiesterase 4D), 1UHO (phosphodiesterase 5), and 1N51 (aminopeptidase P). Of these, cyclin dependent kinase 2 has a clear role in cancer cell proliferation [13].

A second classification model was constructed using the pseudomolecule data and leave-many-out cross-validation. For this model, precision on the positive labels was 0.78 and precision on the negative labels was 0.66. The difference in prediction accuracy between the docking-data and pseudomolecule models was not significant based on McNemar's test (*p *= 0.62).

To determine if the leave-many-out models were finding spurious relationships, each was compared to three models that used scrambled synergism scores. To conservatively account for family-wise errors in McNemar's test, the Bonferroni adjustment suggests that an *α *of 0.017 be used rather than 0.05 for determining significance (this is based on two families of three comparisons each). The *p*-values for the docking-data models were 1.0E-07, 0.012, and 2.1E-6. The *p*-values for the pseudomolecule data were 1.3E-04, 1.1E-05, and 5.0E-07. Thus for both models, *p*-values indicated that scrambling the observations produced results incompatible with a null hypothesis that scrambled and non-scrambled models were identical.

The average precision for the scrambled docking-data models was 0.59 and 0.32 on the positive and negative labels, respectively. The average precision on the scrambled pseudo-molecule models was 0.55 and 0.39 on the positive and negative labels, respectively. The precision was not close to 0.5 on the scrambled models because the data sets were unbalanced, and in addition the training algorithm favored models that exhibited similar precision for positive and negative labels.

The precision values on scrambled responses, 0.59 and 0.32, is lower than the precision on nonscrambled responses, 0.77 and 0.60, for non-synergistic and synergistic classes, respectively. This suggests, for example, that if 1,000 mixtures were suitably modeled, and model precision did not change, the scrambled response model would generate 1.8 times more false positives (287 vs. 161) and 1.7 times more false negatives (204 vs. 120) than the model with nonscrambled responses.

Leave-one-out cross-validation was also conducted for the docking-data and pseudomolecule models. Because leave-one-out CV training sets contained 44 rather than 26 mixtures, and training set mixtures included all drugs, it was expected that precision would be higher in the leave-one-out models. Indeed, precision on the negative labels (the smaller and more difficult group to predict) was markedly improved for both the docking-data and pseudomolecule models. For the docking-data model, precision was 0.73 and 0.80 on the positive and negative labels, respectively. For the pseudomolecule model, precision was also 0.73 and 0.80 on the positive and negative labels, respectively.

### Additional model validation based on 10 new samples

To provide an additional test set, 10 new mixtures were tested in the laboratory. A regression model based on the 45 core mixtures was constructed that used binary indicators of mixture composition and docking data as explanatory variables. Using this model, predictions were made for all 1,013 mixtures. Ten of the mixtures predicted to be most synergistic for different mixture sizes were selected for additional laboratory assessment. Predictions made by the regression model are not discussed in detail here, as the model itself was only modestly accurate as demonstrated by leave-one-out cross validation (the *r*^2 ^was 0.45). In effect, the regression model was only used to identify 10 mixtures for additional testing.

Results from the laboratory analysis suggested that five of the mixtures were highly synergistic. Observed synergism scores are listed in Table S.3 of Additional File 1, along with predictions made by the regression model. The docking-data classification model, which was trained on the 45 mixtures, was used to make predictions on these 10 new mixtures. Precision was 0.80 (four of five predicted correctly) and 1.0 (five of five predicted correctly) on the positive and negative labels, respectively.

### Dose reduction through use of mixtures

**Figure 1:**
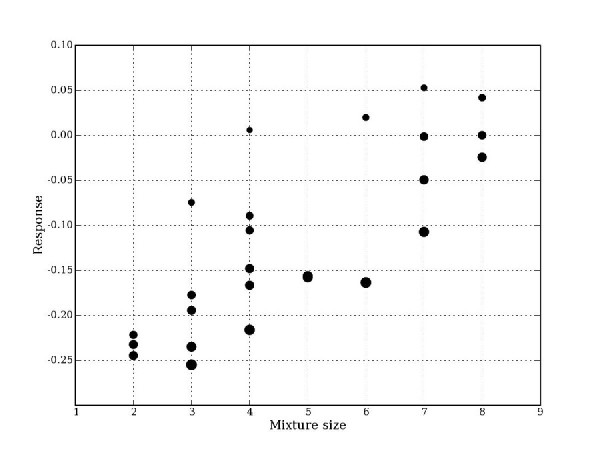
**Doxorubicin dose reduction vs. mixture size and observed response for 25 doxorubicin-containing mixtures.** The relative degree of dose reduction is indicated by marker size. The smallest circle corresponds to a doxorubicin dose reduction of 3.30 and the largest circle to a dose reduction of 10.87. Dose reduction is calculated from experimental data.

The degree of synergism may not be the best index for identifying promising mixtures. An alternative index is the degree of dose reduction that can be achieved for a given drug. For example, one of the dose-limiting side effects of doxorubicin is cardiac toxicity [14]. To prevent this, mixtures could be chosen to minimize the dose of doxorubicin required for a given effect level. Based on the experimental data listed in Tables S.1 and S.2, doxorubicin dose-reduction values for the 25 doxorubicin-containing mixtures tested are plotted in Figure 1 against the number of drugs per mixture and observed synergism score. The mixture with the greatest dose reduction was M47, which contained doxorubicin, curcumin, and juglone. The IC50 of doxorubicin alone was 5.22 μL and that of M47 was 12.36 μL. The fraction of doxorubicin in the mixture was 0.039. Therefore, M47 allowed a 5.22/(12.36·0.039) = 10.9-fold reduction in doxorubicin concentration to achieve the same effect (50 percent inhibition) as doxorubicin used alone. Some larger mixtures also showed high dose reduction, even though they were less synergistic. For example, dose reduction values for M49 and M50, which had five and six components, respectively, were 9.1 and 10.8, respectively. The dose reduction value for M35, with seven drugs, was 9.7.

## Discussion

Mixtures can be designed to produce a wide range of effects. The work in this paper focused on cytotoxicity, but other possibilities include inhibition of invasion, metastasis, angiogenesis, drug resistance, or any combinations of these. All processes present worthwhile targets for drug therapy and ideally, mixtures to affect them would be rationally designed. Such a design could be based on protein-drug affinity and the topology and dynamics of the protein-protein and signaling networks involved. Much of the needed network and affinity data are not yet available, however, and therefore the approach taken in this paper is much more modest. Here, mixtures are modeled using virtual docking data as a surrogate for binding affinity values.

As previously noted, the degree to which virtual docking data reflects binding affinity is uncertain for the compounds modeled here. Thus, while the binding data can be viewed as useful mathematical descriptors for discriminating between highly synergistic and modest or non-synergistic mixtures, the data cannot be confidently interpreted in a biologic sense. In the future, as virtual docking programs become more accurate, the method proposed here could lend itself to biologic interpretation. In this sense, the proposed method has greater potential than the pseudomolecule approach.

If biologic interpretations were to be made, several issues would remain to be addressed. For example, does the drug in fact enter the cancer cell at sufficient concentrations and in an active biologic form that is similar to the one used in virtual docking? Is synergism against cancer cells likely to be greater than synergism against normal cells? In addition, care must be taken in assessing the feature selection choices. Real-valued docking scores were transformed into binary scores and these scores were transformed into counts. The loss in numerical diversity resulted in high correlations between the docking data for some proteins. The average squared correlation coefficient between the 45-element docking-data vectors (count data) of different proteins was 0.31 (stdev = 0.26). Of the 286 vectors, 179 duplicates occurred, leaving 107 unique vectors. Therefore, a particular choice by the feature selection algorithm would also implicate any other proteins that have highly correlated (or identical) scores. Training on a larger data set could reduce the number of duplicates.

While much work would remain to demonstrate that any of the mixtures studied here are clinically useful, the reported results do have an immediate value. They suggest that reasonably accurate predictive models of drug synergism could be constructed using relatively small training sets, and that the models could have sufficient generalizability to allow predictions on mixtures that contain drugs the model had not been trained on. This means, potentially, that promising mixtures created from drug libraries could be identified after sampling only a small fraction of possible mixtures. Training and testing sets larger than the ones used here may be desirable, however, as they might improve precision and aid in model assessment.

When constructing models, training sets should be selected to adequately sample the space of explanatory variables that is of interest. The classification models were able to accurately predict which mixtures that contain a new, unseen drug would be highly synergistic, except when that drug was doxorubicin. For the doxorubicin CV test set, the precision on negative labels was only 0.08. The low precision can be explained by the fact that doxorubicin is quite different from the other drugs studied, both in structure and effect. For example, it was more cytotoxic and its binary protein docking scores were different than other drugs. The average squared correlation coefficient of 286-element binary docking score vectors between doxorubicin and other drugs was 0.006, compared with a mean of 0.07 for that of all other drugs. The correlation for doxorubicin was markedly lower than that for any other single drug. To obtain accurate predictions for doxorubicin, it would be necessary to train the model using mixtures that contained drugs somewhat similar to doxorubicin. Doxorubicin itself, or its minor variations, would not necessarily be needed, however. Thus, while the leave-many-out model was not able to accurately predict the synergism class for doxorubicin-containing mixtures, the leave-one-out model was able to do so. (Doxorubicin-containing mixtures were included in the leave-one-out training sets.) Also, precision in the leave-many-out model for doxorubicin mixtures could likely be increased by including additional drugs in the training set that are similar to doxorubicin.

When identifying promising mixtures, the potential for dose reduction may be an important characteristic to consider. As shown in Figure 1, dose reduction for doxorubicin can be increased both by increasing synergism and by increasing the number of drugs in a mixture. The ability to target multiple proteins is also a characteristic worth considering. Larger mixtures may therefore have advantages even if they afforded slightly less dose reduction than smaller, more synergistic ones. While increasing the number of drugs could increase the risk of adverse effects, that risk may be minimized if a low dose of each individual compound is used (as could be the case in larger mixtures) and if several of the drugs in a mixture are relatively non-toxic (as was the case for the nine natural compounds tested here).

Many other characteristics of drugs and mixtures that are important in mixture design are not addressed here. For example, the toxicity patterns of component drugs are important. In general, mixtures will show lower systemic toxicity if the organ toxicity patterns of individual drugs do not overlap. The pharmacokinetic properties of component drugs in a mixture are also important, as useful plasma concentrations of each drug must be achieved. Investigations of these and other topics remain for future work.

## Conclusion

There is need within the drug development and toxicology fields for accurate, predictive models of drug interaction. The models proposed here suggest that synergism can be predicted and that measures of protein-drug virtual docking can be useful as explanatory variables. Cross-validation results presented here suggest that the docking-data approach may be useful, even when training sets derive from a small fraction of the sample space. Experiments conducted here identified synergistic mixtures, some of which could allow substantially lower doxorubicin concentrations without a reduction in *in-vitro *efficacy.

## Methods

### Selection of compounds

Doxorubicin was selected as a typical chemotherapy drug because it is water-soluble and it was adequately cytotoxic against the H460 human lung cancer cell line in the 48-hour assay used here. It has been used clinically against small cell [15] and non-small cell [16] lung cancer, as well as a variety of other cancers including breast [17] and ovarian [18]. Vitamin K3, juglone, quercetin, luteolin, baicalein, epigallocatechin gallate (EGCG), plumbagin, and rhein were selected from a set of 115,000 natural compounds using predictions from two QSAR models [19]. The QSAR models identified several dozen compounds that were commercially available, predicted to be modestly to strongly cytotoxic *in-vitro *against three cell lines used in the NCI screening program (H460, MCF7, and SF-268) [20], and predicted to have low rat LD50 values (low systemic toxicity). Of these, 22 were tested in-vitro and the 8 listed above were sufficiently water-soluble and cytotoxic in the 48-hour assay to allow their use. Curcumin was included based on reports of its activity against the H460 cell line [21], its reported safety [22], and its activity in the assay used here.

### Mixture composition

Out of the 1,013 possible mixtures, 45 were selected for testing using a semi-random process where the average mixture size was designed to be between three and four drugs, each new mixture was chosen to be maximally different from all previously constructed ones, and all drugs were used in a roughly equal number of mixtures. Relative concentrations between drugs in a mixture were set at a fixed ratio based on the IC50 of each drug (as measured in μL, see below). The composition of individual mixtures is given in Table S.2 of Additional File 1.

### Drug storage and modification of solubility

To maintain consistent drug concentrations between in-vitro testing rounds, solutions for all drugs except EGCG and doxorubicin were prepared once and then frozen in aliquots. These eight drugs were mixed with cyclodextrin to improve water solubility. Approximately 50 mg of each drug was mixed with twice its weight of hydroxypropyl beta-cyclodextrin obtained from Cyclodextrin Technologies Development, Inc. (High Springs, FL) and added to phosphate-buffered saline to make a 4.0 mg/ml solution (of drug). Solutions of all eight drugs except juglone and plumbagin were briefly heated to boiling to increase solubility and then passed through a 0.45-micron filter to remove undissolved drug. Solutions of juglone and plumbagin were heated to 46 degrees centigrade and then filtered. After filtering, solutions were aliquoted and frozen at -20 degrees centigrade until use. To test each drug or mixture, enough of each solution was thawed for treating three replicate trays. Solutions were warmed and then again passed though a 0.45-micron filter to remove any drug that may have precipitated during freezing or thawing. The filtered solutions, termed *stock solutions*, were combined to create the needed mixtures, which were used within four days. Drug concentrations in the stock solutions were assayed by HPLC as described below. EGCG and doxorubicin were sufficiently water-soluble and did not require cyclodextrin. Fresh stock solutions of these two drugs (at 10 mg/ml and 500 μg/ml, respectively, in phosphate-buffered saline) were prepared each time a new batch of aliquots was thawed.

Drug and mixture concentrations are reported in units of μL per well times 20, unless otherwise stated. To calculate the drug concentration in μg/ml that is equivalent to a concentration given in μL, multiply the concentration of the stock solution by the concentration in μL and divide by 4 ml. For example, a 3.5 μL concentration of EGCG is equivalent to (10 mg/ml)(3.5 μL)(1/4 ml) = 8.75 μg/ml.

### Cytotoxicity assays

The human non-small cell lung cancer line H460 was obtained from American Type Culture Collection (ATCC, Manassas, VA) and cultured using RPMI 1640 media as recommended by ATCC, supplemented with 10 percent fetal calf serum. Doxorubicin was obtained from Bedford Laboratories (Bedford, OH). Curcumin, vitamin K3, juglone, quercetin, luteolin, baicalein, EGCG, plumbagin, and rhein were obtained from Sigma-Aldrich (St. Louis, MO).

The *in-vitro *growth inhibitory effects of all drugs and mixtures were assessed using the CellTiter-Blue assay purchased from Promega (Madison, WI). It is a fluorometric assay for estimating the number of viable cells based on reduction of an indicator dye, resazurin, by living cells. Briefly, approximately 600 cells in 100 μL media were seeded in each well of 96-well microtiter trays and incubated in a humidified atmosphere of five percent CO_2 _overnight. Wells were then treated with various drug concentrations in media (total volume 200 μL per well). Control wells were given media instead of drug solution. After 48 hours incubation, 10 μL reagent was added to each well and trays were incubated for an additional three hours. Fluorescence was read using a Flex800 microplate reader at an excitation wavelength of 540 nm and emission wavelength of 590 nm (BioTek, Winooski, Vermont). Fluorescence values for each drug concentration (including zero drug concentration) were normalized using results obtained from wells treated solely with media, drug, and assay reagent. Each assay was performed at least in triplicate. To reduce testing time, a 48-hour assay was used rather than the more typical 72-hour assay. All drugs used here produced complete or near complete cell kill at high drug concentrations in the 48-hour assay. Three trays were treated only with beta-cyclodextrin solution to assess its toxicity. Results suggested that the cyclodextrin concentrations used in the mixtures were not cytotoxic.

### HPLC assessment of drug concentrations

The HPLC system consisted of a Waters (Milford, MA) Separations Module 2695 and Waters Photodiode Array detector 996, acquiring from 200 to 500 nm. The column was a Phenomenex (Torrance, CA) Luna 5u, C18, 250 × 4.60 mm. The mobile phase consisted of a binary solvent system of 0.5 percent formic acid in water (solvent A) and methanol (solvent B). The flow rate was 1 ml/min. Compounds were eluted at a linear gradient (50 percent B from 0 to 1 min; 50–95 percent B from 1 to 15 min; 95 percent B from 15 to 20 min; 95-50 percent B from 20 to 21 min; and 50 percent B from 21 to 25 min). Waters Empower Pro software was used to collect and analyze data. Calibration was performed using results from triplicate analysis of serial dilutions of pure compounds. Retention times, wavelengths, and calculated drug concentrations in the stock solutions are summarized in Table S.4 of Additional File 1. Doxorubicin and EGCG were highly water-soluble and so concentrations did not need to be measured by HPLC. To obtain the concentration in μg/ml for a drug concentration reported in μL, multiply the drug concentration in the stock solution (from Table S.4, Additional File 1) by the concentration in μL and divide by 4 ml. To obtain the total drug concentration in a mixture, use a weighted average of drug concentrations in stock solutions, with fractions of drugs in the mixture used as weights.

### Assessment of drug interactions by the MixLow method

The MixLow method [3] (and Boik J, Narasimhan B: Introducing the R package mixlow for assessment of drug synergism/antagonism, submitted) was used to assess drug interactions based on results from the cytotoxicity assays. In brief, the MixLow method utilizes a nonlinear mixed-effects model to accurately estimate parameters of sigmoidal concentration-effect curves. The parameter estimates are used to construct an estimator of a Loewe-additivity interaction index, ${\hat{L}}_{\phi }$. Index estimates are obtained at various fraction affected (*φ*) values, where fraction affected refers to the expected fraction of the cell population affected by a given drug concentration. An interaction index of 1.0 at a specified fraction affected indicates additivity, an index less than 1.0 indicates synergism, and an index greater than 1.0 indicates antagonism. A short mathematical explanation of the MixLow method and the Loewe index is provided in Additional File 1.

**Figure 2:**
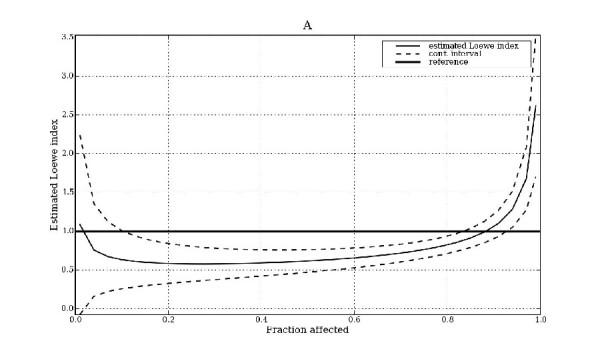
**Example for mixture M15.** Fraction affected vs. estimated Loewe index, with confidence intervals.

An example of fraction affects vs. estimated interaction index is given in Figure 2 for the mixture M15. Ninety-five percent confidence intervals of the index are also shown (dotted lines). Statistically significant synergism is indicated for this mixture between 0.10 ≤ *φ *≤ 0.85 (where both confidence intervals are less than 1.0) and antagonism is indicated at a fraction affected greater than about 0.93 (where both intervals are greater than 1.0). Additivity is indicated at a fraction affected less than 0.1 (where the confidence intervals span 1.0).

The responses (synergism scores) modeled in this paper were constructed as an area under the curve of statistically significant antagonism minus the area under the curve of statistically significant synergism. Index values over the interval 0.31 ≤ *φ *≤ 0.64 were used. For example, in Figure 2 both confidence intervals are below 1.0 in this interval. Thus the area under the curve of statistically significant antagonism is zero and that for statistically significant synergism is the area between the upper confidence interval and the reference line of 1.0 over this *φ *interval. The calculated response would thus be negative, indicating that on average there was more synergism than antagonism occurring. A calculated response of zero would indicate that no significant synergism or antagonism is occurring (i.e., additivity cannot be ruled out over this *φ *interval) or that antagonism and synergism are occurring but are balanced. The interval 0.31 ≤ *φ *≤ 0.64 was used because lower values of *φ *would not have a large impact on cell viability and higher values of *φ *may require excessive drug concentrations (greater than might be achieved clinically). Moreover, confidence intervals are typically wider at extreme *φ *values. Preliminary models that used the interaction index itself as the response variable were less accurate than ones based on confidence intervals (data not shown). Programming of the MixLow method was done in the R [23] and Python (with Numpy/Scipy) [24,25] environments.

### Selection of proteins and virtual docking

Because the functions of some proteins are uncertain, and crystal structures are not available for all proteins, it was not possible to conduct virtual docking for every protein that might play a role in drug synergism. Instead, data for a large and diverse subset of proteins was collected. Crystal structures of proteins and their ligands were obtained from the Protein Data Bank [26]. Initially, 46,623 records were downloaded that contained 7,809 unique proteins, each record with a co-crystalized ligand and resolution less than 3.0 angstroms. This set was further refined by removing any record with ligands containing atoms other than C, N, H, O, F, Cl, or S; removing any records with ligands on an exclude list (for example, SO_4 _and ligands with a molecular weight greater than 900 or less than 50). Proteins were also removed if they were from a species other than *Homo sapiens *or *Escherichia coli*, or whose biological detail field contained words on an exclude list (for example, "renin", "renal", "coagulation", and "alcohol"). This filtering process resulted in a set of 1,886 unique proteins, which were then virtually docked with their co-crystalized ligands using the commercial program eHits [7].

EHits is an automated system that requires little user intervention for preprocessing of proteins. It conducts automatic pocket detection on the protein surface, automatic splitting of the ligand from the co-crystalized receptor, automatic assignment of partial charges to atoms, automatic determination of hydrogen protonation states, and automatic correction for common PDB file format errors. Moreover, its scoring function takes advantage of temperature factor information provided in PDB files. eHits was able to successfully dock co-crystalized ligands for 1,087 (Additional File 2), which were then docked with all 10 drugs. Successful docking means that no fatal occurred. Each protein-drug pair was assigned a value of one if the lowest docking score was less than -3.0 and less than the docking score for the co-crystalized ligand plus 0.1. Otherwise, the protein-drug pair was assigned a value of zero. In this way, only poses with a low score relative to those for all ligands and proteins and a score lower than or approximately equal to the co-crystalized ligand was considered as a hit. There were 286 proteins for which at least one drug was assigned a value of one. This list of 286 proteins is provided in Table S.5 of Additional File 1. The mixture-protein score was obtained by counting the number of drugs in a mixture that were predicted to bind to a given protein (i.e., that had a protein-drug assignment of one). This resulted in a 45 × 286 matrix of mixture-protein scores (Additional File 3), which is referred to in the text as *docking data*. The term *features *is used to refer to columns of explanatory variables, whether from mixture composition, structural descriptors, or docking data.

### Generation of structural descriptors

The commercial program Dragon [27] was used to generate 1,664 molecular descriptors for use in testing the pseudomolecule approach to modeling mixture interactions. Low-energy conformers of three-dimensional drug structures were obtained using molecular mechanics. Duplicate, constant, and completely correlated descriptors were removed, leaving approximately 1,200 descriptors for modeling, depending on the training set. All used descriptors were standardized to mean zero and unit standard deviation. No attempt was made to presuppose the relative importance of individual descriptors. Descriptors for each mixture were obtained by averaging the descriptors over all component drugs. A weighted average of descriptors with weights based on percent of mixture content produced models of lower performance (data not shown).

### KMLA

Regression and classification models were built using KMLA (kernel multitask latent analysis), an approach developed by Xiang and Bennett [28] based on earlier work by Momma and Bennett [29] and used here with minor changes [19]. Briefly, KMLA is closely related to partial least squares (PLS) [30] and can be used for many of the same problems, although it has several distinct differences. KMLA allows multiple tasks to be learned, nonlinear relationships to be modeled, and arbitrary loss functions to be used. PLS and related algorithms allow use of highly correlated explanatory variables, as well as large number of explanatory variables relative to the number of records. For these reasons, they are often used with microarray data where the number of explanatory variables exceeds the number of records [31]. Here, KMLA was used in single-task mode. The KMLA algorithm was coded to allow both regression and classification. A mathematical explanation of the KMLA algorithm is provided in [19].

### Responses for classification models

To construct responses for classification models, the most synergistic 30 percent of drugs were assigned the label -1 and the remaining 70 percent were assigned the label +1. Therefore, the training sets were unbalanced. To help assure that equal accuracy was obtained for both labels, a cost was assigned in the training algorithm to misclassified negative labels in proportion to the fraction of negative labels.

### Model selection

To use the KMLA algorithm, the number of latent features must be specified. Because models were constructed using 45 mixtures, common sense would suggest that no more than a few latent features would be appropriate. Use of too many latent features could be expected to degrade the ability of the model to generalize to new data. In this paper, two latent features were used for all models constructed. This choice was determined from training set results – for all training sets the third latent feature provided little additional gain in training set accuracy.

The kernel type and any associated kernel parameters also must be specified. A Gaussian kernel function is employed for all models constructed here, as is common in kernel regression and classification problems. The Gaussian kernel has one parameter that must be chosen, kernel width (*σ*^2^). Because very few training samples are available relative to the number of explanatory variables, it could be expected that a linear or near-linear kernel would produce the best results. Here a near-linear kernel was constructed by setting the width parameter to 5,000, a very high value. Model accuracy was not very sensitive to modest variations in kernel width (data not shown).

Lastly, when used for classification the KMLA algorithm requires that a threshold parameter be specified for separating classes. This parameter was chosen based on training set results as further described in [19].

### Feature selection

To improve the accuracy of regression and classification models, an iterative backwards elimination feature selection algorithm was used. As noted above, the number of features available for the pseudomolecule models was approximately 1,200. As with the Dragon data, duplicate, constant, and completely correlated descriptors were also removed from the docking data and then the remaining descriptors were standardized to mean zero and standard deviation one. Out of the 286 docking data features, 107 were unique. Of these, approximately 90 remained unique after partitioning into training/testing sets for cross validation.

In each iteration features were removed that did not contribute greatly to predictions. More specifically, in each iteration a model was constructed using a data set of *m *features and *n *rows, and predictions were made for the training set. In the first iteration, *m *equaled the total number of available features (approximately 1200 for the pseudomolecule models and approximately 90 for the docking data models). Four models were created, where the number of retained latent features in each was one, two, three, and four. Thus, four predictions were made for each training point and predictions formed a matrix $\hat{Y}\in R^{n\times 4}$, where *n *is the number of training examples (44 for the leave-one-out models). Next, *m *additional $\hat{Y}$ matrices were produced, each one for a data set where one of the *m *features was omitted. The score for the *i*th feature was calculated as $S_{i}=\left\|{\hat{Y}}_{m}-{\hat{Y}}_{-i}\right\|$, where the subscript *m *refers to use of all available features and the subscript *-i *refers to use of all available features except feature *i*. If removal of feature *i *did not alter the predictions at all, the score *S*_*i *_would be equal to zero. Features with a score less than 30 percent of the maximum score for that round were removed and a new iteration was started using the reduced feature set. No more than 15 percent of the available features were removed in any single iteration. The iterations continued until the scores for all remaining features were greater than 30 percent of the maximum score for that round. Feature selection was conducted using all data for a given model. For example, if the model was constructed using both binary indicators of mixture composition and docking data, feature selection was done on the combined data set.

### Model validation

Leave-one-out and leave-many-out cross validation was used to validate the classification models. The mixtures that were set aside in a given cross-validation round (CV test sets) represented a true hold-out test set. Each cross-validation round employed its own feature selection process. In this way, feature selection was conducted without knowledge of the hold-out mixtures. Similarly, model training occurred without knowledge of the hold-out mixtures. During data preprocessing for each round, removal of duplicate features, centering of features, and scaling of features by their standard deviations occurred after partitioning the data set, and so also occurred without knowledge of the hold-out mixtures.

The leave-many-out procedure consisted of 10 outer rounds, one for each drug. In each outer round, all mixtures containing that drug were placed in the hold-out set. Typically, these hold-out sets contained 19 mixtures and the model was trained on 26 mixtures. In this way, the model was validated using a set of mixtures such that each mixture contained a drug that the model had not been trained on. Within each outer round, a cross-validation procedure was used whereby the training set was partitioned into 10 verification sets. When the classification models were used to make predictions on new data, predictions on the 10 inner-round training sets were averaged.

Models were also assessed by a standard leave-one-out cross-validation procedure. While leave-one-out procedures are approximately unbiased for the true prediction error, they can have high variance because the CV training sets can be so similar to one another [32]. On the other hand, leave-many-out procedures can have lower variance but greater bias, especially if training sets are small. For the small data sets used here, leave-one-out cross validation provides a reasonable complement to the leave-many-out procedure.

Precision is reported for the classification models. Precision on the positive labels (sensitivity) is defined as the number of records that are experimental positives and predicted to be positive, divided by the total number of experimental positives. Precision on the negative labels (specificity) is defined as the number of records that are experimental negatives and predicted to be negative, divided by the total number of experimental negatives. Note that experimental positives and experimental negatives refer to the synergistic activity of mixtures, as determined in the laboratory.

To compare classifiers, McNemar's test was used as suggested by Dietterich [33]. In brief, a confusion matrix was constructed based on results from leave-many-out cross-validation. Let *N*_*A *_refer to the number of examples classified correctly by classifier *A *but not by classifier *B*, and let *N*_*B *_refer to the number correctly classified by *B *but not *A*. The statistic

$$\frac{{\left(\left|N_{A}-N_{b}\right|-1\right)}^{2}}{N_{A}+N_{b}}$$

is distributed (approximately) as *χ*^2 ^with one degree of freedom.

Lastly, classification models were validated by a Y-randomization test in which a set of models was constructed using scrambled ordering of the responses.

## Authors' contributions

JCB developed the modeling approach, coded the software, and was the primary author of the manuscript. RAN reviewed the study design, participated in coordination of the study, and helped draft the manuscript.

## Supplementary Material

Additional File 1 Supplemental Information. Tables of data and a summary of the MixLow method. Click here for file

Additional File 2 eHits docking scores for 1087 proteins. eHits docking scores for 1087 proteins Click here for file

Additional File 3 docking data for 286 proteins. docking data for 286 proteins. Click here for file
