# Supplementary material for: A classification model to predict synergism/antagonism of cytotoxic mixtures using protein-drug docking scores
Source: BMC Pharmacol. 2008 Jul 29;8:13. doi: 10.1186/1471-2210-8-13 (PMC2526994; doi:10.1186/1471-2210-8-13)
Supplement: Additional File 1 — Supplemental Information. Tables of data and a summary of the MixLow method. [file 1471-2210-8-13-S1.pdf]

## Supplemental Information

Data for the initial 45 mixtures tested are listed in Table S.1. The column *Observed Responses* lists synergism scores.

**Table S.1 . Data for the initial 45 mixtures**

| Drug/ Mixture | Log IC50 (μL) | SE Log IC50 (μL) | IC50 (μL) | Observed Responses | Cross-Validation Predictions | Doxorubicin Dose Reduction | Number of Drugs |
|---|---|---|---|---|---|---|---|
| Baicalein | 3.33 | 0.11 | 28.04 (0.75 μg/ml) | – | – | – | 1 |
| Curcumin | 4.44 | 0.03 | 85.18 (1.71 μg/ml) | – | – | – | 1 |
| Doxorubicin | 1.65 | 0.15 | 5.22 (0.65 μg/ml) | – | – | – | 1 |
| EGCG | 3.55 | 0.05 | 34.67 (86.68 μg/ml) | – | – | – | 1 |
| Juglone | 4.40 | 0.03 | 81.35 (1.59 μg/ml) | – | – | – | 1 |
| Luteolin | 4.00 | 0.06 | 54.35 (4.10 μg/ml) | – | – | – | 1 |
| Plumbagin | 3.83 | 0.03 | 45.90 (1.49 μg/ml) | – | – | – | 1 |
| Quercetin | 3.83 | 0.08 | 46.17 (4.16 μg/ml) | – | – | – | 1 |
| Rhein | 3.92 | 0.05 | 50.55 (7.79 μg/ml) | – | – | – | 1 |
| Vitamin K3 | 4.35 | 0.02 | 77.48 (10.38 μg/ml) | – | – | – | 1 |
| M1 | 4.58 | 0.04 | 97.43 | 0.07 | 0.31 | 4.01 | 10 |
| M2 | 4.39 | 0.03 | 80.53 | 0.20 | 0.15 | – | 6 |
| M3 | 4.16 | 0.04 | 63.83 | -0.07 | -0.06 | – | 2 |
| M4 | 1.42 | 0.11 | 4.12 | -0.20 | -0.24 | 7.40 | 2 |
| M5 | 4.05 | 0.07 | 57.48 | 0.00 | 0.01 | – | 2 |
| M6 | 3.77 | 0.05 | 43.25 | 0.04 | -0.02 | – | 3 |
| M7 | 4.48 | 0.06 | 88.53 | 0.06 | 0.31 | – | 2 |
| M8 | 3.55 | 0.07 | 34.96 | 0.00 | 0.00 | 6.71 | 7 |
| M9 | 4.81 | 0.04 | 122.95 | 0.07 | 0.39 | – | 4 |
| M10 | 4.13 | 0.14 | 61.90 | 0.07 | 0.05 | 3.98 | 7 |

| | | | | | | | |
|---|---|---|---|---|---|---|---|
| M11 | 3.82 | 0.04 | 45.56 | -0.01 | -0.07 | – | 2 |
| M12 | 4.13 | 0.03 | 61.94 | 0.04 | 0.09 | – | 2 |
| M13 | 3.95 | 0.06 | 51.92 | -0.05 | 0.02 | – | 2 |
| M14 | 4.41 | 0.04 | 81.95 | 0.01 | 0.11 | – | 5 |
| M15 | 3.04 | 0.10 | 20.93 | -0.09 | -0.07 | 4.16 | 3 |
| M16 | 3.89 | 0.02 | 49.13 | 0.12 | 0.00 | – | 2 |
| M17 | 4.31 | 0.05 | 74.70 | 0.14 | 0.03 | – | 5 |
| M18 | 4.23 | 0.09 | 68.88 | 0.04 | 0.18 | – | 2 |
| M19 | 3.69 | 0.07 | 40.22 | 0.13 | -0.02 | 8.08 | 8 |
| M20 | 2.99 | 0.10 | 19.92 | -0.25 | -0.18 | 6.62 | 3 |
| M21 | 2.98 | 0.10 | 19.62 | -0.11 | -0.16 | 9.36 | 5 |
| M22 | 3.59 | 0.05 | 36.11 | 0.01 | 0.00 | – | 2 |
| M23 | 4.06 | 0.05 | 58.24 | 0.06 | 0.04 | 5.09 | 8 |
| M24 | 4.06 | 0.05 | 58.24 | -0.05 | 0.02 | 4.29 | 6 |
| M25 | 1.38 | 0.14 | 3.97 | -0.12 | -0.23 | 7.35 | 2 |
| M26 | 2.60 | 0.13 | 13.51 | -0.14 | -0.19 | 7.36 | 3 |
| M27 | 4.53 | 0.03 | 92.82 | 0.05 | 0.08 | – | 3 |
| M28 | 4.20 | 0.03 | 66.47 | 0.00 | 0.00 | – | 2 |
| M29 | 3.58 | 0.06 | 36.04 | -0.12 | 0.01 | 3.30 | 4 |
| M30 | 2.96 | 0.09 | 19.28 | -0.02 | -0.09 | 5.46 | 4 |
| M31 | 4.06 | 0.03 | 58.13 | 0.18 | 0.01 | – | 5 |
| M32 | 3.88 | 0.06 | 48.66 | 0.08 | -0.02 | – | 3 |
| M33 | 4.16 | 0.05 | 64.06 | 0.18 | 0.09 | – | 5 |
| M34 | 2.92 | 0.08 | 18.53 | -0.11 | -0.17 | 7.82 | 4 |
| M35 | 3.36 | 0.08 | 28.80 | -0.11 | -0.11 | 9.74 | 7 |
| M36 | 4.14 | 0.05 | 63.08 | -0.04 | 0.01 | – | 2 |
| M37 | 2.83 | 0.10 | 16.95 | -0.07 | -0.11 | 6.21 | 4 |
| M38 | 4.36 | 0.03 | 78.39 | 0.04 | 0.15 | – | 4 |
| M39 | 3.93 | 0.04 | 50.68 | 0.03 | 0.02 | – | 3 |
| M40 | 4.30 | 0.04 | 73.76 | -0.03 | 0.01 | – | 2 |
| M41 | 4.13 | 0.05 | 62.29 | 0.03 | 0.00 | – | 5 |
| M42 | 3.82 | 0.05 | 45.73 | -0.01 | -0.05 | – | 2 |
| M43 | 2.05 | 0.08 | 7.79 | -0.18 | -0.23 | 9.13 | 3 |
| M44 | 4.35 | 0.04 | 77.27 | 0.24 | 0.09 | – | 6 |
| M45 | 2.77 | 0.15 | 15.92 | -0.15 | -0.15 | 7.68 | 4 |

Table S.2 provides information on the composition of mixtures tested. For modeling purposes, the mixture composition value for a given drug was assigned the value one if the fraction of drug in the mixture was greater than zero and assigned the value zero otherwise. Except for counts of drugs, values listed in the table are fractions of the total mixture content.

**Table S.2. Mixture composition for 55 mixtures**

| Drug/ Mixture | Number of Drugs | Baicalein | Curcumin | Doxorubicin | EGCG | Juglone | Vitamin K3 | Luteolin | Plumbagin | Quercetin | Rhein |
|---|---|---|---|---|---|---|---|---|---|---|---|
| Baicalein | 1 | 1.00 | – | – | – | – | – | – | – | – | – |
| Curcumin | 1 | – | 1.00 | – | – | – | – | – | – | – | – |
| Doxorubicin | 1 | – | - | 1.00 | – | – | – | – | – | – | – |
| EGCG | 1 | – | – | – | 1.00 | – | – | – | – | – | – |
| Juglone | 1 | – | – | – | – | 1.00 | – | – | – | – | – |
| Vitamin K3 | 1 | – | – | – | – | – | 1.00 | – | – | – | – |
| Luteolin | 1 | – | – | – | – | – | – | 1.00 | – | – | – |
| Plumbagin | 1 | – | – | – | – | – | – | – | 1.00 | – | – |
| Quercetin | 1 | – | – | – | – | – | – | – | – | 1.00 | – |
| Rhein | 1 | – | – | – | – | – | – | – | – | – | 1.00 |
| M1 | 10 | 0.06 | 0.18 | 0.01 | 0.06 | 0.15 | 0.14 | 0.11 | 0.09 | 0.09 | 0.10 |
| M2 | 6 | 0.10 | 0.28 | – | 0.10 | – | 0.22 | 0.16 | 0.13 | – | – |
| M3 | 2 | – | 0.55 | – | – | 0.45 | – | – | – | – | – |
| M4 | 2 | – | – | 0.17 | 0.83 | – | – | – | – | – | – |
| M5 | 2 | – | – | – | – | – | – | – | – | 0.47 | 0.53 |
| M6 | 3 | 0.19 | – | – | – | 0.48 | – | – | – | – | 0.33 |
| M7 | 2 | 0.37 | – | – | – | – | – | 0.63 | – | – | – |
| M8 | 7 | 0.10 | 0.30 | 0.02 | 0.11 | – | – | – | 0.14 | 0.15 | 0.17 |
| M9 | 4 | – | – | – | 0.16 | 0.37 | – | 0.26 | – | 0.22 | – |
| M10 | 7 | 0.10 | – | 0.02 | 0.10 | 0.24 | 0.23 | 0.17 | – | 0.14 | – |
| M11 | 2 | – | – | – | – | – | 0.63 | – | 0.37 | – | – |
| M12 | 2 | – | – | – | – | – | – | – | 0.49 | 0.51 | – |
| M13 | 2 | – | – | – | 0.38 | – | – | – | – | – | 0.62 |
| M14 | 5 | – | – | – | 0.12 | 0.27 | 0.26 | – | 0.16 | – | 0.19 |
| M15 | 3 | – | – | 0.06 | – | – | – | 0.47 | – | – | 0.47 |
| M16 | 2 | – | – | – | – | – | – | 0.55 | 0.45 | – | – |
| M17 | 5 | – | 0.29 | – | – | – | 0.23 | 0.17 | – | 0.15 | 0.17 |
| M18 | 2 | 0.40 | – | – | – | – | – | – | – | 0.60 | – |
| M19 | 8 | 0.07 | 0.22 | 0.02 | – | 0.18 | 0.17 | 0.13 | 0.10 | 0.11 | – |
| M20 | 3 | – | 0.53 | 0.04 | – | – | 0.43 | – | – | – | – |
| M21 | 5 | 0.13 | 0.38 | 0.03 | 0.14 | 0.32 | – | – | – | – | – |
| M22 | 2 | – | – | – | 0.43 | – | – | – | 0.57 | – | – |

| | | | | | | | | | | | |
|---|---|---|---|---|---|---|---|---|---|---|---|
| M23 | 8 | – | – | 0.02 | 0.09 | 0.20 | 0.19 | 0.14 | 0.11 | 0.12 | 0.14 |
| M24 | 6 | – | 0.28 | 0.02 | – | 0.23 | – | 0.17 | 0.13 | – | 0.16 |
| M25 | 2 | 0.82 | – | 0.18 | – | – | – | – | – | – | – |
| M26 | 3 | – | – | 0.05 | – | 0.59 | – | – | – | 0.36 | – |
| M27 | 3 | – | – | – | – | 0.38 | 0.36 | 0.26 | – | – | – |
| M28 | 2 | – | – | – | – | 0.64 | – | – | 0.36 | – | – |
| M29 | 4 | 0.20 | – | 0.04 | – | – | 0.47 | – | 0.28 | – | – |
| M30 | 4 | – | – | 0.05 | 0.24 | – | – | 0.39 | 0.32 | – | – |
| M31 | 5 | 0.11 | – | – | – | 0.27 | 0.25 | 0.19 | – | – | 0.18 |
| M32 | 3 | – | 0.51 | – | 0.18 | – | – | 0.30 | – | – | – |
| M33 | 5 | 0.14 | – | – | 0.14 | 0.33 | – | – | 0.19 | 0.20 | – |
| M34 | 4 | – | 0.49 | 0.04 | – | – | – | – | 0.23 | 0.25 | – |
| M35 | 7 | 0.09 | 0.25 | 0.02 | 0.09 | 0.21 | 0.20 | – | – | – | 0.14 |
| M36 | 2 | – | – | – | 0.30 | 0.70 | – | – | – | – | – |
| M37 | 4 | 0.23 | – | 0.05 | – | – | – | – | – | 0.34 | 0.39 |
| M38 | 4 | 0.14 | 0.42 | – | – | – | – | – | 0.20 | – | 0.24 |
| M39 | 3 | 0.23 | – | – | 0.24 | – | 0.53 | – | – | – | – |
| M40 | 2 | – | 0.66 | – | – | – | – | – | – | 0.34 | – |
| M41 | 5 | – | 0.31 | – | 0.11 | 0.25 | – | – | – | 0.15 | 0.18 |
| M42 | 2 | – | – | – | – | – | 0.61 | – | – | 0.39 | – |
| M43 | 3 | – | – | 0.07 | 0.36 | – | – | – | – | – | 0.57 |
| M44 | 6 | 0.09 | 0.26 | – | – | 0.22 | – | 0.15 | – | 0.13 | 0.15 |
| M45 | 4 | – | – | 0.04 | 0.21 | – | 0.46 | – | – | 0.29 | – |
| M46 | 2 | – | 0.93 | 0.07 | – | – | – | – | – | – | – |
| M47 | 3 | – | 0.52 | 0.04 | – | 0.44 | – | – | – | – | – |
| M48 | 4 | – | 0.37 | 0.03 | – | 0.31 | 0.29 | – | – | – | – |
| M49 | 5 | – | 0.30 | 0.02 | – | 0.25 | 0.24 | – | – | – | 0.18 |
| M50 | 6 | – | 0.27 | 0.02 | 0.10 | 0.23 | 0.22 | – | – | – | 0.16 |
| M51 | 7 | – | 0.24 | 0.02 | 0.09 | 0.20 | 0.19 | – | – | 0.12 | 0.14 |
| M52 | 8 | – | 0.22 | 0.02 | 0.08 | 0.18 | 0.17 | – | 0.10 | 0.11 | 0.12 |
| M53 | 3 | – | 0.38 | – | – | 0.32 | 0.30 | – | – | – | – |
| M54 | 4 | – | 0.31 | – | – | 0.26 | 0.25 | – | – | – | 0.18 |
| M55 | 5 | – | 0.28 | – | 0.10 | 0.23 | 0.22 | – | – | – | 0.16 |

Data for the 10 additional mixtures tested are listed in Table S.3.

**Table S.3. Data for 10 additional mixtures**

| Drug/ Mixture | Log IC50 (µL) | SE Log IC50 (µL) | IC50 (µL) | Observed Responses | Predicted Responses | Doxorubicin Dose Reduction | Number of Drugs |
|---|---|---|---|---|---|---|---|
| M46 | 2.50 | 0.07 | 12.23 | -0.22 | -0.24 | 6.19 | 2 |
| M47 | 2.51 | 0.07 | 12.36 | -0.26 | -0.22 | 10.87 | 3 |
| M48 | 2.93 | 0.07 | 18.80 | -0.22 | -0.22 | 10.13 | 4 |
| M49 | 3.23 | 0.09 | 25.30 | -0.16 | -0.20 | 9.14 | 5 |
| M50 | 3.17 | 0.08 | 23.86 | -0.16 | -0.19 | 10.75 | 6 |
| M51 | 3.60 | 0.09 | 36.52 | -0.05 | -0.13 | 7.99 | 7 |
| M52 | 3.89 | 0.06 | 48.68 | 0.00 | -0.08 | 6.69 | 8 |
| M53 | 4.85 | 0.03 | 127.59 | 0.17 | -0.06 | - | 3 |
| M54 | 4.91 | 0.02 | 135.81 | 0.28 | -0.04 | - | 4 |
| M55 | 4.40 | 0.05 | 81.62 | 0.04 | -0.03 | - | 5 |

Data for the drug concentrations in stock solutions are listed in Table S.4.

**Table S.4. Drug concentration in stock solutions**

| Drug | Wavelength (nm) | Retention time (min) | Drug concentration in stock solution (μg/ml) |
|---|---|---|---|
| Baicalein | 276 | 12.6 | 107 |
| Curcumin | 424 | 14.9 | 80 |
| Doxorubicin | – | – | 500 |
| EGCG | – | – | 10,000 |
| Juglone | 251 | 11.2 | 78 |
| Luteolin | 351 | 10.9 | 302 |
| Plumbagin | 266 | 14.2 | 130 |
| Quercetin | 255 | 10.2 | 360 |
| Rhein | 258 | 17.1 | 616 |
| Vitamin K3 | 250 | 12.4 | 536 |

Out of the 7,809 unique proteins downloaded from the Protein Data Bank (PDB) and discussed in the text, 286 were successfully docked to their ligands using eHits and were found to bind with at least one of the 10 drugs used. These proteins are listed in Table S.5.

**Table S.5. List of PDB proteins used in models**

| | | | | | | | | |
|---|---|---|---|---|---|---|---|---|
| 1N51 | 1QJ3 | 1SWX | 2GJ6 | 1EEF | 1JH9 | 1VJA | 1Z57 | 2ANO |
| 1NAI | 1QP0 | 1T4V | 2GPP | 1EFI | 1JQE | 1WSS | 1ZD5 | 2AOU |
| 1NHT | 1QP7 | 1TA6 | 2H42 | 1EK4 | 1JTQ | 1WSV | 1ZFK | 2AOV |
| 1O5F | 1QPZ | 1TB7 | 2H44 | 1EUY | 1JWT | 1X2H | 1ZGE | 2AWH |
| 1OOQ | 1QQB | 1TF0 | 2HBY | 1F4E | 1JYX | 1X76 | 1ZGF | 2AX6 |
| 1ORK | 1QXK | 1TGV | 2HU6 | 1F4F | 1K3L | 1X7R | 1ZGY | 2B1V |
| 1OSF | 1R0P | 1TKY | 2IMG | 1F91 | 1K3Y | 1X97 | 1ZHM | 2B50 |
| 1OTY | 1R1J | 1TR7 | 2IPW | 1FJ4 | 1K5Q | 1XF0 | 1ZHP | 2B52 |
| 1OUM | 1R1X | 1TRG | 2IPX | 1FQG | 1K5S | 1XGJ | 1ZHR | 2B7D |
| 1OV4 | 1R55 | 1TT6 | 2IS7 | 1FYF | 1K97 | 1XM4 | 1ZJ2 | 2BAL |
| 1OV6 | 1R9O | 1TZ8 | 2ITM | 1G1B | 1KCE | 1XMU | 1ZJ3 | 2BDG |
| 1OYN | 1RBQ | 1U1C | 2J1N | 1G27 | 1KE9 | 1XMY | 1ZJP | 2BH3 |
| 1P57 | 1RC2 | 1U3Q | 2PUE | 1G81 | 1KEC | 1XNZ | 1ZKN | 2D0T |
| 1P60 | 1RHU | 1U3U | 2TSC | 1GM8 | 1KKB | 1XON | 1ZLT | 2D5Z |
| 1P61 | 1RKP | 1U3V | 4PRG | 1GSJ | 1KMN | 1XOQ | 1ZML | 2DN1 |
| 1PF8 | 1RMT | 1U3W | 1AHF | 1HDX | 1KQU | 1XOS | 1ZRK | 2ETK |
| 1PG2 | 1RMY | 1U6Q | 1AI4 | 1HO5 | 1KRU | 1XWK | 1ZYS | 2ETR |
| 1PJ2 | 1RMZ | 1U71 | 1AIB | 1I2Z | 1KSN | 1Y2C | 1ZZ2 | 2EU2 |
| 1PJ4 | 1RO6 | 1U72 | 1AIQ | 1I30 | 1KW0 | 1Y2D | 2A0W | 2EVT |
| 1PMV | 1ROS | 1U9E | 1AJN | 1ICR | 1L6Y | 1Y2J | 2A0X | 2EXM |
| 1PNR | 1RRM | 1UHO | 1AJP | 1ICU | 1LI4 | 1Y8J | 2A0Y | 2F6W |
| 1PVS | 1RSZ | 1UKI | 1AMR | 1IF4 | 1LLB | 1YCI | 2A2Q | 2F7I |
| 1PWM | 1RT9 | 1UWF | 1AMS | 1IF5 | 1LO6 | 1YDB | 2A2R | |
| 1PWY | 1RWK | 17GS | 1AN5 | 1IF6 | 1LT8 | 1YDK | 2A2S | |
| 1PXI | 1RWO | 19GS | 1AXW | 1IF7 | 1M9M | 1YKR | 2A4Z | |
| 1PXJ | 1RWW | 20GS | 1AZ1 | 1IKT | 1M9Q | 1YMX | 2A5U | |
| 1PXK | 1S14 | 2FAI | 1BDH | 1IQG | 1MD3 | 1YOE | 2A8U | |
| 1PYE | 1S1D | 2FES | 1BJ0 | 1IQN | 1MD4 | 1YOL | 2AAC | |
| 1Q0B | 1S8C | 2FHY | 1BQ1 | 1IRJ | 1MLW | 1YPJ | 2AB6 | |
| 1Q3A | 1SB1 | 2FLS | 1DAH | 1J7E | 1MMK | 1YRY | 2AER | |
| 1Q4N | 1SC8 | 2FTO | 1DDU | 1J99 | 1MMT | 1YTA | 2AGT | |
| 1Q5K | 1SD1 | 2G8O | 1DJR | 1JFT | 1MUO | 1YXX | 2AI8 | |
| 1Q6K | 1SQT | 2GG3 | 1DNP | 1JH1 | 1VJ9 | 1Z11 | 2ANM | |

**Short mathematical description of the MixLow method**

In typical *in-vitro* cytotoxicity experiments, responses are measured at different drug concentrations. Such studies commonly utilize multi-well incubation trays, where groups of wells within each tray receive different drug concentrations, and control wells receive no drug treatment. To assess drug interactions in a mixture, four steps are typically followed:

1. Individual drugs and the mixture are assayed for cytotoxicity against a target cancer cell line.
2. Parameters of the concentration-response curves are estimated.
3. The estimated parameters are used in a null interaction model to estimate drug interaction. For the MixLow method, the null interaction model is based on Loewe additivity. The Loewe additivity index produces the intuitively reasonable result that a *sham* mixture, a mixture of a drug with itself, is additive.
4. Confidence intervals of the index are calculated.

The MixLow method [1] uses a nonlinear mixed-effects model to estimate parameters of the concentration-effect curves. Random effects are commonly associated with observations sharing the same level of a classification factor, and in the case of the MixLow method this classification factor is tray. The MixLow method can be used to quantify drug interactions in any fixed-ratio drug combination study that includes within-group and between-group replicates, and where responses follow a sigmoidal pattern.

Let the random variable $Fa$ signify the fraction of cells affected by a drug concentration. Define $\phi = \mathrm{E}\left[Fa\right]$, where $\mathrm{E}[\bullet]$ is the expected value. In some contexts $\phi$ is estimated based on concentration-response data and in other contexts a concentration is estimated that results in a fixed value of $\phi$. Denote by $\psi_{d,\phi}$ the $\phi$-effective log concentration of drug *d*. This is the log concentration that produces a fraction affected equal to a fixed $\phi$. For example, the log concentration of drug *d* that inhibits proliferation of a cell population by 10 percent relative to controls is denoted by $\psi_{d,0.1}$. By convention, $\exp(\psi_{d,0.1})$ is called the IC10 (10 percent inhibitory concentration).

The Loewe index provides a measure of drug interaction. For two drugs, the Loewe index and its estimator[1] are

$$L_\phi = \sum_{d=1}^{2} \frac{\exp(m_{d,\phi})}{\exp(\psi_{d,\phi})} \text{ and } \hat{L}_\phi = \sum_{d=1}^{2} \frac{\exp(\hat{m}_{d,\phi})}{\exp(\hat{\psi}_{d,\phi})}, \qquad (1)$$

respectively, where $m_{d,\phi}$ is an unknown constant signifying the log concentration of drug *d* in the mixture when the mixture is at its $\phi$-effective log concentration, and $\psi_{d,\phi}$ is the unknown $\phi$-effective log concentration of drug *d* alone. The mixture is synergistic,

[1] The hat notation is used here to denote parameter estimators and estimates.

additive, or antagonistic at $\phi$ depending on whether the value of the Loewe index is less than 1, equal to 1, or greater than 1, respectively.

The MixLow model uses a nonlinear mixed-effects framework to represent the concentration-response curve. As a skeleton description, responses are modeled as the expected mean of control wells times a sigmoidal function, plus an error term. Sigmoidal models of this type are sometimes referred to as Hill models. Formally, responses, $\{Y_{d,t,w}\}$, obtained from unprocessed data are modeled as a sigmoidal function of the concentration:

$$Y_{d,t,w} = \exp(\mu + b_t)(1-\phi_{d,t,w}) + \varepsilon_{d,t,w}, \text{ where} \tag{2}$$

$$\phi_{d,t,w} = 1 - \frac{1}{1+\left(\dfrac{\exp(c_{d,t,w})}{\exp(\psi_{d,0.5})}\right)^{\gamma_d}} \tag{3}$$

and the subscripts *d,t,w* refer to the *d*th drug, *t*th tray, and *w*th well, respectively. Here, drug *d* could refer to a single drug or a mixture, and $c_{d,t,w}$ refers to the log of the drug concentration for the *d,t,w*th observation, a known constant. The expected value of $\exp(\mu + b_t)$ refers to the expected mean of control wells from all trays, where $b_t$ is a random deviate specific to tray *t*. The notation (1-$\phi$) is used to denote the expected fraction unaffected, rather than introducing a new symbol for the latter. The exponent term in Model (2) is used to ensure that the expected response in control wells is always positive.

Values $\{b_t\}$ are independently distributed as $b_t \sim \text{N}(0,\sigma_b^2)$. The error terms in Model (2) are independently distributed as $\varepsilon_{d,t,w} \sim \text{N}\left(0, f(\sigma^2, \text{E}\left[Y_{d,t,w} \mid b_t\right])\right)$, where $f(\bullet)$ is an error function such as $\sigma \text{E}\left[Y_{d,t,w} \mid b_t\right]^{\beta_d}$. Here, the power parameter, $\beta_d$, is drug-dependent.

Discussions on implementation of the method, modifications to Model (2), and procedures used to calculate confidence intervals of the interaction index are provided in Boik et al. [1].

## References

1. Boik J, Newman R, Boik R: **Quantifying synergism/antagonism using nonlinear mixed-effects modeling: A simulation study**. *Statistics in Medicine,* 2008. **27**(7):1040-61.
